# Ibuprofen Would Be the First-Line Nonsteroidal Anti-inflammatory Drug for Polymyalgia Rheumatica: A Case Series of Five Patients

**DOI:** 10.7759/cureus.58778

**Published:** 2024-04-22

**Authors:** Shigeko Inokuma, Taro Okazaki, Hiroki Morishita, Masanori Tsuji, Yoshimasa Goto

**Affiliations:** 1 Department of Allergy and Rheumatism, Chiba Central Medical Center, Chiba-shi, JPN; 2 Department of Rheumatism and Collagen Diseases, Kohnodai Hospital, National Center for Global Health and Medicine, Ichikawa-shi, JPN; 3 Department of Internal Medicine, Chiba Central Medical Center, Chiba-shi, JPN

**Keywords:** methotrexate, elderly, steroid, nonsteroidal anti-inflammatory drug, ibuprofen, polymyalgia rheumatica

## Abstract

The primary treatment of choice for polymyalgia rheumatica (PMR) is corticosteroids, which are better avoided for elderly patients susceptible to PMR. The cases of five patients cured with only a small dosage of 600 mg/day ibuprofen without steroids or methotrexate are reported. Their clinical features were compared with those of the 26 PMR patients who had steroids and/or methotrexate in addition to ibuprofen.

PMR was diagnosed based on the 2015 EULAR/ACR criteria. They were all females aged 73-80. They all had no giant cell arteritis or autoantibodies. Nonsteroidal anti-inflammatory drugs (NSAIDs) other than ibuprofen had not worked in four cases; for the one, ibuprofen was the first NSAID. Their serum CRP levels were 1.57-12.8 mg/dL at ibuprofen introduction. Colchicine was co-administered in two patients. At the next visit three to seven days after ibuprofen introduction, they all showed a clear recovery with a CRP level decrease. Ibuprofen tapering was started within three months, and no relapse was until two to five years’ follow-up. Comparison with the 26 patients who had additional steroid and/or methotrexate showed that the disease duration until ibuprofen introduction was statistically significantly shorter in the five patients (1.40±0.65 vs 3.28±2.98 months). Ibuprofen would be the first-line drug for PMR, and its earliest use would be beneficial.

## Introduction

Recently, as many countries are experiencing aging in their societies, the incidence rates of diseases peculiar to the elderly have been increasing, including polymyalgia rheumatica (PMR) [1]. The first-line therapy for PMR is corticosteroids (steroids) [2]. Although steroids are better avoided for elderly patients susceptible to PMR, long-term use of over two years was reported in half of the patients, and the relapse rate at one year was over 40% [3]. Much stronger therapies with biological disease-modifying anti-rheumatic drugs have now been under evaluation, whereas none of the nonsteroidal anti-inflammatory drugs (NSAIDs) are recommended and any have been disregarded [2].

We successfully treated five PMR patients with ibuprofen at a substantially small dosage of 600 mg/day without either steroid or methotrexate administration. Two out of the five had concomitant colchicine, and one had it after a clear recovery. These cases of patients in whom ibuprofen worked were examined by comparing their features with those of patients who had steroids or methotrexate in addition to ibuprofen. The use of a known drug with fewer adverse events might be useful and help look into the pathology.

## Case presentation

**Figure 1:**
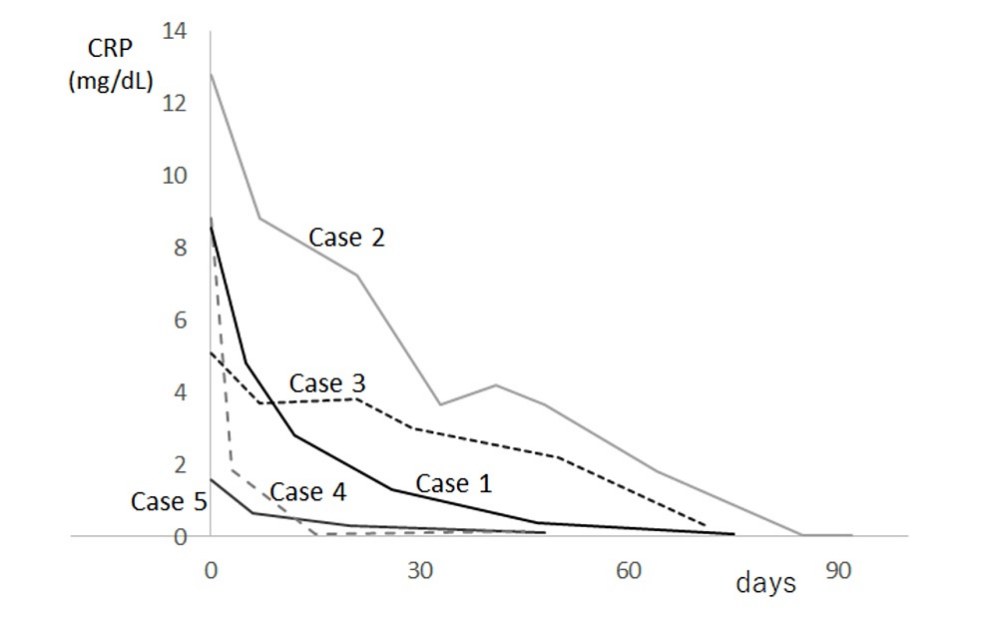
Serum CRP level changes under ibuprofen therapy Ibuprofen introduction was on Day 0.

During the five years from August 2015 to July 2020, 43 patients (28 females and 15 males, 74.6±4.5 years old) were diagnosed as having PMR on the basis of ACR/EULAR criteria in our hospital [2]. Among them, 31 patients had 600 mg/day ibuprofen, and five out of the 31 recovered without steroids or methotrexate (MTX). Two among the five had concomitant colchicine. The treatment prior to ibuprofen introduction and the changes in laboratory data are summarized in the table including their physiques. After ibuprofen withdrawal, no recurrence was until 2023. Written informed consent was obtained from the five patients. Their clinical courses are summarized in Table 1 and Figure 1.

**Table 1:** Patients who recovered from polymyalgia rheumatica with 600 mg/day ibuprofen ibu, ibuprofen therapy; (days), days after ibu introduction; NA, not available.

						CRP (mg/dL)			ESR (mm/h)			Albumin (g/dL)		Hb (g/dL)	
		Age	Height	Weight	Therapy	At ibu start	At next visit after ibu	At ibu tapering start	At ibu start	At next visit after ibu	At ibu tapering start	At ibu start	At ibu tapering start	At ibu start	At ibu tapering start
Case	Sex	(yr)	(cm)	(kg)	Prior to ibu										
1	F	76	144	47	loxoprofen diclofenac pregabalin	8.54	4.82 (5 days)	0.08 (75 days)	130	130	28	2.3	4	8.9	11.3
																2	F	78	146	46	loxoprofen nerve block	12.8	8.81 (14 days)	0.02 (85 days)	115	105	11	2.4	3.6	9.3	11.7
																																3	F	80	148	42	celecoxib intra-knee steroid	5.1	3.7 (7 days)	0.32 (71 days)	NA	71	14	2.8	3.3	9.1	10.4
4	F	73	153	53	none	8.81	1.85 (3 days)	0.08 (15 days)	127	127	63	3.4	4.4	12.4	14.4																																
																5	F	76	152	48	loxoprofen acetaminophen	1.57	0.65 (7 days)	0.11 (47 days)	27	33	12	3.5	4.2	10.9	11.9

Case 1

**Figure 2:**
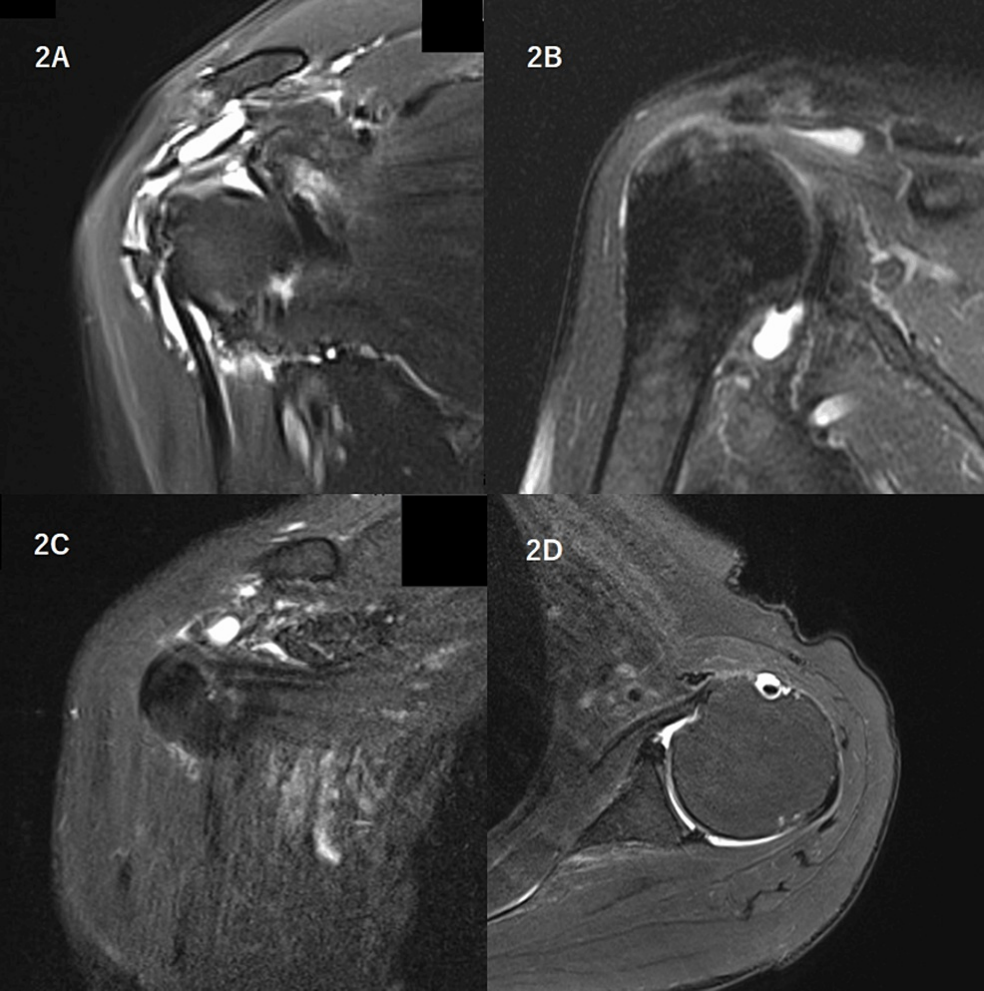
Magnetic resonance imaging. (A) Case 1: inflamed tissues around right shoulder, (B) Case 2: fluid in subacromial bursa, (C) Case 4: fluid in bursa at ventral side of acromioclavicular joint, (D) Case 5: fluid in bursa of long head of biceps brachii. MRI was not obtained for Case 3.

A 76-year-old female patient presented with one-month-long pain and stiffness around her shoulder and hip areas in May 2017. Standing up from the sitting position was impossible for her. Magnetic resonance imaging (MRI) showed inflamed tissues around the shoulder (Figure 2A).

Loxoprofen or diclofenac had not ameliorated her pain. Five days after taking ibuprofen and colchicine (1.0 mg/day), her pain disappeared, and she achieved the full range of motion of her shoulders and hips. Within one month, her daily activities returned to normal. Her CRP level became normal within 15 days after the ibuprofen introduction. Thereafter, her ESR steadily decreased to normal until two and a half months later. Colchicine was discontinued and ibuprofen was tapered off. Five years later, she was well with no recurrence.

Case 2 

A 78-year-old female patient had suffered from pain in her shoulder and hip areas for one and a half months in July 2018. Loxoprofen was ineffective. She could not raise her upper limbs and had edema in her extremities. MRI showed fluid retention in the subacromial bursa (Figure 2B), and echography did similar findings in the long tendon sheaths of the biceps brachii and the subdeltoid and subacromial bursae. Ibuprofen relieved her pain in one week with a CRP level decrease. On the other hand, her ESR remained high, and her albumin and hemoglobin levels further decreased to 2.3 g/dL and 8.3 g/dL, respectively. Six weeks later, when her CRP level had already decreased from 12.8 to 1.81 mg/dL, 1.5 mg of colchicine was added. After achieving relief, both drugs were tapered off within six months. She had been well for four years after their withdrawal.

Case 3 

An 80-year-old female patient had in difficulty standing and walking, could not go up the stairs, and had edema in her extremities for two months in July 2020. She had lost 6 kg of weight. MRI was not taken. Ibuprofen and 1 mg of colchicine ameliorated her pain within a week. She had been well with no symptoms during her four-year follow-up with both drugs tapered off.

Case 4

A 73-year-old female patient could not get up from her bed one morning owing to pain in her shoulder and hip areas in July 2020. Until the previous evening, she was able to carry out her usual daily activities. At her first visit two weeks later, she could not raise her upper arms. MRI showed fluid retention in the bursa at the ventral side of the acromioclavicular joint (Figure 2C). Ibuprofen was the first medication prescribed. The following day after taking ibuprofen, her symptoms improved. Three days later, her pain almost completely disappeared with a marked CRP level decrease from 8.81 to 1.85 mg/dL. Two weeks later, she was completely well. Her CRP level became normal within 15 days after the ibuprofen introduction. Thereafter, her ESR decreased. A three-year follow-up after withdrawing ibuprofen showed no relapse.

Case 5

A 76-year-old female patient could not get out of the car after a three-hour ride one day in February 2020 because of shoulder and pelvic area pain. Loxoprofen did not relieve her pain. Two months later, an MRI showed fluid in the bursa around her shoulder (Figure 2D). Five days after ibuprofen treatment, her pain disappeared. In six weeks, she was able to perform her usual daily activities. She had been well for three years after the withdrawal.

All of the patients had neither apparent arthritis, fever, nor signs of crowned dens syndrome. Ophthalmic and ultrasound examinations showed no signs of temporal arteritis. They were negative for autoantibodies (RF, anti-CCP antibody, ANA, anti-SS-A antibody, anti-SS-B antibody, MPO-ANCA, and PR3-ANCA). Their white blood cell counts and levels of creatinine and liver enzymes were within the normal ranges.

In Cases 1-3 and 5, NSAIDs prescribed by the previous doctors had been ineffective (Table). After taking ibuprofen, they showed a clear subjective improvement and chose to maintain the same regimen. Their serum CRP levels clearly decreased at their next visits, three to seven days after ibuprofen introduction (Fig.1), whereas their ESRs remained high (Table). Thereafter, both steadily decreased with clear increases in their hemoglobin and serum albumin levels (Table).

None of them concomitantly took systemic steroids, methotrexate (MTX), or biological drugs. Colchicine was co-administered with ibuprofen in Cases 1 and 3. In Case 2, colchicine was added after a clear recovery had been achieved. The ibuprofen dosage was tapered off without any recurrence until three to five years later. No adverse event was observed in any of the five cases.

Comparison of features between cases 1-5 and the patients who had additional steroids and/or MTX

Cases 1-5 were among the 31 PMR patients who were prescribed ibuprofen. The daily dosage was 600 mg. None had had ibuprofen therapy before visiting us. Twenty-six patients (17 females and nine males) except for Cases 1-5 had additional steroids and/or MTX. Among the 26, 25 had a steroid. The remaining patient who had no steroid was a 78-year-old female with three-month-long upper arm pain and walking disability. She had diabetes mellitus and was introduced early to MTX, which was 20 days after the ibuprofen prescription when her CRP level had decreased from 13.7 mg/dL at the first visit to 7.73 mg/dL, whereas ESR had from 120 mm/h to 114 mm/h. MTX, once a week, was stopped after four doses because of the elevation of liver enzyme levels, and she recovered with ibuprofen alone, from which she was ultimately withdrawn. Including this patient, 14 out of the 26 had MTX.

Comparison of the features between Cases 1-5 and the remaining 26 patients showed that sex or age (75.0±1.7 vs 77.5±4.9 years old) did not differ significantly nor did the CRP level (7.36±3.79 vs 8.28±4.84 mg/dL). However, the duration from symptom onset to ibuprofen introduction was significantly shorter in Cases 1-5 than in the 26 patients (1.40±0.65 vs 3.28±2.98 months, p<0.05 using the unpaired t-test).

## Discussion

Most worth noting in this study is that only a substantially small dosage of ibuprofen cured PMR in five patients. Other NSAIDs had been ineffective in all but one for whom ibuprofen was the first medication. In addition, it was found that the earliest introduction elicited a good response.

Studies on the prevalence of PMR are few, but the number of PMR patients is expected to increase with the aging of society [1,4-6]. The diagnostic criteria of Bird et al. included age 65 years or more [7], and the criteria of EULAR/ACR included age 50 years or older, but PMR occurs with peak incidence at 70 to 75 years of age [8]. Recent imaging studies have added bursitis in the shoulder or pelvic girdles as a criterion [2,9]. The five cases were clearly diagnosed with PMR, and other diagnoses including pseudogout were not likely.

Conventionally, the initial therapy has always been steroids, and their long-term administration is the current mainstay [4]. For patients with a poor response, a relapsing disease, or a relative contraindication, MTX or biologic therapies including anti-IL-6 antibodies are recommended [10], although these therapies and steroids should best be avoided for the elderly.

NSAIDs are presently not among the treatment choices. Even when NSAIDs are prescribed, ibuprofen is not among them, maybe because it is a cheap over-the-counter drug considered to have insufficient anti-inflammatory or anti-analgesic effects. None of the previous doctors of our patients prescribed ibuprofen. After visiting us, ibuprofen was adopted during the phase until laboratory and imaging test results were known. Regarding why ibuprofen was adopted in our cases, one is that previous NSAIDs including loxoprofen, diclofenac, and celecoxib had been ineffective. Another was the experience in Case 1, in whom prompt subjective recovery was obtained with a clear CRP level decrease in only five days. This success led the doctors in charge to choose ibuprofen for other cases as well. Moreover, we have previously reported at domestic academic meetings an exceptional response to ibuprofen in some auto-inflammatory disease statuses.

The clinical course of Case 4 appeared most outstanding. Two weeks after the disease onset, ibuprofen was the first and only regimen adopted. A marked improvement was obtained in only three days. Ibuprofen introduction was much earlier in this patient. Though the disease duration until ibuprofen introduction varied among Cases 1-5, it was significantly shorter than that of the 26 patients who had steroid and/or MTX additionally. An early introduction would have been related to their good response. In the five cases, their CRP levels clearly decreased in only a few or several days, while their ESRs remained high and decreased much later, indicating that the disease had persisted for a certain duration. Although not significant, CRP level was higher in the 26 patients than in the five cases, which might be due to the longer disease durations in these 26. If ibuprofen had been introduced earlier in the 26 patients, a better response could have been obtained at least in some of them. In addition, one patient out of the 26, who had only four doses of MTX, showed a clear recovery under ibuprofen mono-therapy both prior to and after MTX doses.

Ibuprofen is a propionic acid, developed in the 1960s [11]. There may be such an argument that in case ibuprofen works for PMR, it might have been used more. Some reasons might include that a good response to an over-the-counter drug is easily overlooked and that steroids had long been established as the first line. Among NSAIDs, ibuprofen has a unique profile such as the association with meningitis [12]. Recent studies on ibuprofen have revealed new features of anti-leucocyte [13], antitumor [14], and antimicrobial [15] effects as well as anti-inflammatory, antipyretic, and analgesic effects. A study showed that ibuprofen supported macrophage differentiation and T-cell recruitment in a breast cancer model [13]. Another study showed that ibuprofen reduced macrophage infiltration into the acini of a pancreatitis model [16]. Most recently, it has been the focus of attention in relation to COVID-19 [17]. Although no report on ibuprofen for PMR has been found so far, studies focusing on whether ibuprofen may function via a pathway different from those of other NSAIDs are expected.

In Japan, the recommended dosage of ibuprofen listed in the package insert and approved under the National Medical Insurance System is up to 600 mg/day [18]. It is substantially lower than that (400-800 mg, 3-4 times a day) in Western countries [19]. Our five patients were not petite for an average Japanese woman of their age. Even if they are considered petite by Western standards, the dosage is still too small. Nevertheless, a good response was obtained in these patients with relatively severe PMR. If a much higher dosage, as much as that in Western countries, is prescribed, a much better response for many more patients would be expected. 

The contribution of colchicine seemed limited. Colchicine was prescribed concurrently with ibuprofen in Cases 1 and 3, six weeks after Case 2 when her CRP level had already decreased, and not in Cases 4 and 5. As the effectiveness of colchicine for autoinflammatory diseases or calcium pyrophosphate deposition (CPPD) disease is very possible, colchicine for PMR might be better verified. Spontaneous remission was unlikely in any of the cases. The dosing periods of ibuprofen until an obvious recovery were shorter than the disease durations until ibuprofen introduction.

As an early-acting effect of ibuprofen was observed, administration of this drug, at least until the diagnostic test results are available, is recommended. Even if the officially approved dosage in Japan is small, the earliest use may effectively cure PMR. Alternatively, a dosage as large as that in Western countries would be recommended.

The adverse events related to ibuprofen use, which have been reported to the Japanese official agency, Pharmaceuticals and Medical Devices Agency, and listed on its website, are few and not severe [20].

## Conclusions

The cases of five PMR patients who recovered with only a substantially low dosage of ibuprofen were reported. NSAIDs other than ibuprofen used for the four patients had been ineffective; for one patient, ibuprofen was the first NSAID. Their disease durations until ibuprofen introduction were significantly shorter than those of 26 PMR patients who had steroids and/or MTX in addition to ibuprofen. The earliest ibuprofen administration would be recommended for PMR patients.
